# Comparative investigation of respiratory tract involvement in granulomatosis with polyangiitis between PR3-ANCA positive and MPO-ANCA positive cases: a retrospective cohort study

**DOI:** 10.1186/s12890-015-0068-1

**Published:** 2015-07-30

**Authors:** Satoshi Ikeda, Machiko Arita, Kenta Misaki, Yumiko Kashiwagi, Yuhei Ito, Hirotaka Yamada, Machiko Hotta, Akihiro Nishiyama, Akihiro Ito, Maki Noyama, Takashi Koyama, Kenji Notohara, Tadashi Ishida

**Affiliations:** Department of Respiratory Medicine, Kurashiki Central Hospital, Miwa 1-1-1, Kurashiki, 710-8602 Japan; Department of Endocrinology and Rheumatology, Kurashiki Central Hospital, Kurashiki, Okayama Japan; Department of Pathology, Kurashiki Central Hospital, Kurashiki, Okayama Japan; Department of Radiology, Kurashiki Central Hospital, Kurashiki, Okayama Japan

**Keywords:** Granulomatosis with polyangiitis, Respiratory tract involvement, Ant-neutrophil cytoplasmic antibody, Proteinase 3, Myeloperoxidase

## Abstract

**Background:**

The clinical characteristics of myeloperoxidase antineutrophil cytoplasmic antibody (MPO-ANCA) positive granulomatosis with polyangiitis (GPA) remain unclear, as does the difference between MPO-ANCA positive GPA and proteinase 3 (PR3)-ANCA positive GPA, especially with regard to the details of respiratory tract involvement. We investigated the differences in clinical, radiological, and histopathological features between PR3-ANCA positive GPA and MPO-ANCA positive GPA.

**Methods:**

We retrospectively reviewed 16 patients who were newly diagnosed with GPA between December 2000 and July 2014. One patient, who was positive for both PR3-ANCA and MPO-ANCA, was excluded. Our review was based on the European Medicine Agency (EMA) algorithm.

**Results:**

Fifty-six percent of GPA patients were positive for PR3-ANCA, 38 % for MPO-ANCA, and the remaining 6 % for both. The MPO-ANCA positive group included a greater number of females (67 %). There were no statistically significant differences in laboratory data, symptoms and signs, Birmingham Vasculitis Activity Score, or CT findings between the two groups. As for upper respiratory tract involvement, the most common manifestation was paranasal sinusitis, whereas lung nodules were most common as the lower respiratory tract involvement in both groups. Although the combination therapy with prednisone and cyclophosphamide was the most common initial treatment in both groups, the relapse rate in MPO-ANCA positive cases was lower than that of PR3-ANCA positive cases (17 % and 56 %, respectively).

**Conclusion:**

A high prevalence of MPO-ANCA positive GPA was noted. No significant differences in clinico-radiological findings were observed except for the prevalence of relapse between the PR3-ANCA positive cases and MPO-ANCA positive cases, suggesting that the type of ANCA may be of little help in the diagnosis of GPA. Examination for granulomatous findings in the respiratory tract is important, even in MPO-ANCA positive cases. There is a need to accumulate more cases and conduct a further investigation in the future.

## Background

Granulomatosis with polyangiitis (GPA) is a systemic vasculitis syndrome characterized by necrotizing granulomatous inflammation of the respiratory tracts, systemic necrotizing vasculitis, and necrotizing glomerulonephritis [1]. In 1990, the classification criterion, established with the combination of clinical characteristics and pathological characteristics, was reported by the American College of Rheumatology (ACR) [2], and this was followed by the Chapel Hill Consensus Conference (CHCC), wherein the classification was established from a pathological perspective [3]. In 2007, the European Medicine Agency (EMA) proposed a multi-stage classification algorithm of antineutrophil cytoplasmic antibody (ANCA)-associated vasculitis (AAV) and classic polyarteritis nodosa (PN) using ACR classification criteria, CHCC classification, and surrogate markers for vasculitis and ANCA, with the aim of applying the classification in epidemiological researches [4]. The Boards of Directors of ACR, the American Society of Nephrology, and the European League Against Rheumatism (EULAR) recommended that the name be changed from “Wegener’s granulomatosis” to GPA in 2011 [5].

It is estimated that the onset of GPA involves a genetic background with additional environmental factors (*Staphylococcus aureus* infection [6], silica, etc.) that leads to the production of ANCA, resulting in the excessive activation of neutrophils and causing vascular disorders [7]. Of ANCAs, an autoantibody that acts on proteinase 3 (PR3) is specially noted as the factor leading to the onset of GPA. It is thought that neutrophils are activated under the presence of PR3-ANCA, and subsequently, inflammatory cytokine, reactive oxygen, and protease are released from the neutrophils fixated onto the vascular wall, resulting in the onset of vasculitis and granulomatous inflammation [8].

Although PR3-ANCA is known as a disease marker for GPA and myeloperoxidase (MPO)-ANCA is known as a marker for microscopic polyangiitis (MPA) or eosinophilic granulomatosis with polyangiitis (EGPA), the transfer of antibodies has been observed within AAV, and MPO-ANCA positive GPA has been reported. However, few studies exist regarding MPO-ANCA positive GPA. The clinical characteristics of MPO-ANCA positive GPA have not been fully elucidated, nor has the difference between MPO-ANCA positive GPA and PR3-ANCA positive GPA, especially with regard to the details of respiratory tract involvement.

In the present study, we retrospectively reviewed consecutive cases of GPA to document any differences in clinical, radiological, and histopathological features between PR3-ANCA positive cases and MPO-ANCA positive cases, with a particular focus on respiratory tract involvement.

## Methods

### Patients and setting

This retrospective study was performed at Kurashiki central hospital in Kurashiki city, Okayama, Japan. The diagnosis of GPA was based on EMA algorithm. In this algorithm, using the ACR classification criteria, CHCC classification, surrogate markers for vasculitis, and the presence or absence of ANCA, the cases can be classified in EGPA, GPA, MPA, and classic PN. Either PR3 or MPO can be used as correspondent antigen of ANCA. When histopathological investigation cannot be conducted, surrogate markers for granulomatous inflammation and necrotizing glomerulonephritis are used for classification [4]. From December 2000 to July 2014, 16 patients newly diagnosed with GPA based on EMA algorithm were included in the study. One case positive for both PR3-ANCA and MPO-ANCA was excluded from the subsequent comparative investigation of clinical, radiological, and histopathological features because the number of cases is small. The Ethics Committee of Kurashiki Central Hospital approved this study protocol. The Ethics Comitee approved the waiver of each patient’s consent because it was a retrospective study and high anonymity was secured.

### Clinical and laboratory findings

Clinical data and laboratory results were extracted from the patients’ medical records. The factors examined were sex, age, the time from onset to first visit and first visit to treatment start, the department for the first visit, symptoms and signs (according to the items of Birmingham Vasculitis Activity Score; BVAS) at the time of diagnosis, and laboratory data (inflammatory markers, serum creatinine, and urine analysis). Disease activity was assessed by BVAS version 3 [9] at the time of diagnosis.

### Radiological findings

Chest computed tomography (CT) findings were reviewed and interpreted by two pulmonologists (IS, MA) and one radiologist (TK) blinded to the biopsy results and clinical outcomes. The presence, extension, and distribution of the following CT findings were evaluated: small nodules (major axis diameter less than 10 mm), large nodules (10–30 mm), mass (more than 30 mm), consolidation, ground glass opacity, centrilobular nodular shadow, bronchial wall thickening (each levels of trachea, main bronchi, lobar bronchi, and segmental/sub-segmental), thickening of interlobular septa, mediastinal /hilar lymphadenopathy, and pleural effusion.

### Histopathological analysis

Two pathologists reviewed the specimens (nasal mucosa, lung, and kidney) independently, and determined the presence of the following findings according to the items of ACR classification criteria and CHCC classification: (1) granuloma/granulomatous inflammation of an artery or perivascular area, (2) necrotizing vasculitis/glomerulonephritis, and (3) granulomatous inflammation of the respiratory tract. Moreover, cases of glomerulonephritis was classified into the categories of focal, crescentic, mixed, or sclerotic according to the histopathologic classification of ANCA-associated glomerulonephritis proposed by Berden [10].

### Statistical analysis

Categorical data are presented as number (percentage). Continuous data are presented as the median (interquartile range). Fisher’s exact test was used to compare categorical data. Mann–Whitney *U* test was used to compare continuous data. A p value <0.05 was considered statistically significant.

## Results

### Clinical characteristics and laboratory data

**Fig. 1:**
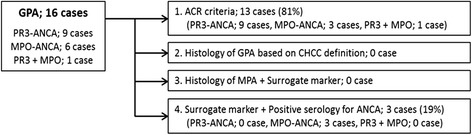
Classification of the patients according to the European Medicine Agency algorithm. According to the EMA algorithm, all PR3-ANCA positive cases, three of six MPO-ANCA positive cases, and one double-positive case met the ACR criteria. The remaining three MPO-ANCA positive cases were diagnosed as GPA on the basis of the surrogate markers and positivity for ANCA without histological proof of granuloma or necrotizing vasculitis. Abbreviations: EMA, European Medicine Agency; ACR, American College of Rheumatology; CHCC, Chapel Hill Consensus Conference

In this study, all the patients diagnosed as GPA by the EMA algorithm were positive for ANCA. Nine patients were positive for PR3-ANCA, six were positive for MPO-ANCA, and the remaining one was positive for both PR3-ANCA and MPO-ANCA. According to the EMA algorithm, all PR3-ANCA positive cases, three of six MPO-ANCA positive cases, and one double positive case met the ACR criteria (Fig. 1). The patients who did not meet the ACR criteria had neither histology of GPA nor MPA compatible with the CHCC definition. The remaining three MPO-ANCA positive cases were diagnosed as GPA on the basis of the surrogate markers and positivity for ANCA without histological proof of granuloma or necrotizing vasculitis.

The median age at the time of diagnosis was 62.0 years for the PR3-ANCA positive group, 64.0 years for the MPO-ANCA positive group, respectively (Table 1). PR3-ANCA positive group showed almost equal number of male and female, whereas MPO-ANCA positive group included a greater number of female (67 %). In the PR3-ANCA positive cases, the time from onset to first visit was shorter than the MPO-ANCA positive cases.

**Table 1:** Summary of the clinical characteristics and laboratory data

	PR3-ANCA	MPO-ANCA	P value
	(N = 9)	(N = 6)	
Age	62.0 (52.0–64.0)	64.0 (57.5–72.0)	0.443
Sex (male/female)	5 / 4	2 / 4	0.608
Time			
from onset to first visit	12.0 (3.00–22.0)	28.5 (21.8–43.5)	0.045
from first visit to treatment	34.0 (20.0–50.0)	31.5 (15.0–63.0)	0.679
Department for the first visit (%)			
Respiratory medicine	3 (33 %)	4 (67 %)	0.329
Rheumatology	3 (33 %)	2 (33 %)	
Nephlorogy	3 (33 %)	0	
Labopratory data			
White Blood Cell (/μL)	10,600 (8,500–12,000)	13,750 (13,025–14,400)	0.099
Neutrophil count (/μL)	8,578 (6,596–9,434)	11,721 (11,138–13,132)	0.077
Lymphocyte count (/μL)	1,148 (794–1,217)	795 (737–1,274)	0.906
Albumin (g/dL)	3.20 (2.60–3.50)	3.15 (2.65–3.58)	1
Creatinine (mg/dL)	0.70 (0.56–3.34)	0.64 (0.48–0.78)	0.48
C-reactive protein (mg/dL)	8.71 (7.89–9.45)	14.3 (8.83–16.7)	0.195
PR3-ANCA	98.0 (45.0–214)	-	NA
MPO-ANCA	-	61.5 (54.3–83.0)	NA

Categorical data are presented as number (percentage), and were analyzed by Fisher’s exact test. Continuous data are presented as the median (interquartile range), and were analyzed by Mann–Whitney *U* test. A p value of <0.05 was considered statistically significant

Abbreviations: *NA* not applicable

No statistically significant differences of laboratory data were observed between the two groups. However, in the MPO-ANCA positive cases, white blood cell count, neutrophil count, and serum C-reactive protein levels tended to be higher than the PR3-ANCA positive cases.

### Symptoms and signs at the time of diagnosis

With regard to the symptoms and signs based on the items of BVAS at the time of diagnosis, no clinically meaningful differences between PR3-ANCA positive cases and MPO-ANCA positive cases were observed (Table 2).

**Table 2:** Symptoms and signs based on the items of BVAS

	PR3-ANCA	MPO-ANCA	P value
	(N = 9)	(N = 6)	
BVAS (total score)	19.0 (12.0–21.0)	16.5 (14.3–20.3)	0.906
General score	2.00 (0.00–2.00)	2.00 (1.25–2.00)	1.00
Myalgia	0	2 (33 %)	0.143
Arthralgia/Arthritis	2 (22 %)	0	0.486
Fever >38 °C	6 (67 %)	4 (67 %)	1.00
Weight loss >2 kg	1 (11 %)	0	1.00
Cutaneous score	0.00 (0.00–0.00)	0.00 (0.00–1.50)	0.351
Skin vasculitis	1 (11 %)	2 (33 %)	0.525
Mucous membranes/eyes score	0.00 (0.00–3.00)	0.00 (0.00–0.00)	0.261
Significant proptosis	1 (11 %)	0	1.00
Scleritis	2 (22 %)	1 (17 %)	1.00
Conjunctivitis	4 (44 %)	0	0.103
ENT score	4.00 (2.00–6.00)	5.50 (1.25–6.00)	0.806
Bloody nasal discharge	6 (67 %)	1 (17 %)	0.119
Paranasal sinus involvement	4 (44 %)	4 (67 %)	0.608
Conductive deafness	3 (33 %)	2 (33 %)	1.00
Sensorineural hearing loss	3 (33 %)	2 (33 %)	1.00
Purulent nasal discharge*	4 (44 %)	0	0.103
Suddle nose*	1 (11 %)	0	1.00
Nasal septum perforation*	1 (11 %)	0	1.00
Exudative otitis media*	3 (33 %)	3 (50 %)	0.622
Chest score	3.00 (3.00–6.00)	4.50 (3.00–6.00)	0.740
Wheeze	0	1 (17 %)	0.400
Nodules or cavities	8 (89 %)	6 (100 %)	1.00
Pleural effusion	0	2 (33 %)	0.143
Infiltrate	4 (44 %)	1 (17 %)	0.580
Alveolar haemorrhage	0	0	NA
Respiratory failure	0	1 (17 %)	0.400
Cough*	2 (22 %)	5 (83 %)	0.0410
Dyspnea*	0	0	NA
Renal score	4.00 (0.00–12.0)	4.00 (1.00–10.0)	0.851
Hypertension	3 (33 %)	3 (50 %)	0.622
Proteinuria >1+	5 (56 %)	2 (33 %)	0.608
Haematuria >10 rbc/hpf	4 (44 %)	2 (33 %)	1.00
Cr 125–249 μmol/L	0	1 (17 %)	0.40
Cr 250–499 μmol/L	2 (22 %)	0	0.486
Cr >500 μmol/L	2 (22 %)	1 (17 %)	1.00
Rise in Cr >30 % or Ccr fall >25 %	4 (44 %)	1 (17 %)	0.58
Hemodialysis*	2 (22 %)	1 (17 %)	1.00
Nervous system score	0.00 (0.00–0.00)	0.00 (0.00–0.00)	0.842
Headache	1 (11 %)	1 (17 %)	1.00
Hypertrophic pachymeningitis*	1 (11 %)	0	1.00
Retro-orbital mass*	2 (22 %)	0	0.486

Birmingham Vasculitis Activity Score were presented as total scores and scores for every internal organ. Categorical data are presented as number (percentage), and were analyzed by Fisher’s exact test. Continuous data are presented as the median (interquartile range), and were analyzed by Mann–Whitney *U* test. A p value of <0.05 was considered statistically significant. *; Symptoms and signs not included in the items of BVAS.

Abbreviations: *BVAS* Birmingham Vasculitis Activity Score; NA, not applicable

As for upper respiratory tract involvement, the most common manifestation was paranasal sinusitis in both groups (44 % in PR3-ANCA positive cases and 67 % in MPO-ANCA positive cases, respectively), followed by exudative otitis media (33 % and 50 %, respectively). As for lower respiratory tract involvement, lung nodules were the most common manifestation (78 % in PR3-ANCA positive cases and 100 % in MPO-ANCA positive cases, respectively).

The median BVAS at the time of diagnosis was 19.0 in PR3-ANCA positive cases and 16.5 in MPO-ANCA positive cases, respectively. Neither total scores of BVAS nor scores for every internal organ differ between PR3-ANCA positive cases and MPO-ANCA positive cases. Among the items of BVAS, pulmonary nodules or masses were the most frequently observed in both groups.

### Radiological findings

Thoracic manifestations were found in all patients (Table 3). In both groups, nodular shadow was observed at the highest incidence (78 % in PR3-ANCA positive cases and 100 % in MPO-ANCA positive cases). Small nodules (<10 mm) were observed in 34 areas in seven patients in the PR3-ANCA positive group and 66 areas in five patients in the MPO-ANCA positive group. Large nodules (≥10 mm, <30 mm) were observed in 12 areas in three patients in the PR3-ANCA positive group and 63 areas in six cases in the MPO-ANCA positive group. There were no significant differences in the median number of small/large nodules per person between PR3-ANCA positive cases and MPO-ANCA positive cases. The incidence of cavitation was 2.0 % (two of 100 total areas) in the small nodules and 6.7 % (five of 75 total areas) in the large nodules.

**Table 3:** Comparison of HRCT findings between PR3-ANCA positive cases and MPO-ANCA positive cases

	PR3-ANCA	MPO-ANCA	P value
	(N = 9)	(N = 6)	
Small nodule (<10 mm)			
Patients (%)	7 (78 %)	5 (83 %)	1.00
Unilateral/Bilateral	2/5	0/5	0.470
Number per person	2.0 (1.5–8.0)	6.0 (5.0–14)	0.142
Cavity	1	1	NA
Large nodule (≥10, <30 mm)			
Patients (%)	3 (33 %)	6 (100 %)	0.0280
Unilateral/Bilateral	0/3	0/6	NA
Number per person	4.0 (3.5–4.5)	3.0 (2.0–4.8)	0.596
Cavity	2	3	NA
Mass (≥30 mm)			
Patients (%)	0	1 (17 %)	0.400
Unilateral/Bilateral	0/0	0/1	NA
Number per person	0	3.0	NA
Cavity	0	2	NA
Consolidation			
Patients (%)	5 (56 %)	1 (17 %)	0.287
Unilateral/Bilateral	2/3	1/0	1.00
Ground glass opacity (%)	2 (22 %)	4 (67 %)	0.136
Centrilobular nodular shadow (%)	2 (22 %)	0	0.486
Bronchial wall thickening	5 (56 %)	4 (67 %)	1.00
Trachea (%)	1 (11 %)	0	1.00
Main bronchi (%)	2 (22 %)	2 (33 %)	1.00
lobar bronchi (%)	4 (44 %)	3 (50 %)	1.00
Segmental bronchi (%)	2 (22 %)	4 (67 %)	0.136
Thickening of interlobular septa (%)	0	2 (33 %)	0.143
Mediastinal/hilar lymphadenopathy (%)	3 (33 %)	3 (50 %)	0.622
Pleural effusion (%)	1 (11 %)	2 (33 %)	0.525

Categorical data are presented as number (percentage), and were analyzed by Fisher’s exact test

The second most common finding was the thickening of tracheal/bronchial walls (56 % in PR3-ANCA positive cases and 67 % in MPO-ANCA positive cases). In PR3-ANCA positive cases, thickening of bronchial wall at the lobar bronchi level was the most common (44 %), whereas in MPO-ANCA positive cases thickening of bronchial wall at the segmental/sub-segmental bronchi level was the most common (67 %).

**Fig. 2:**
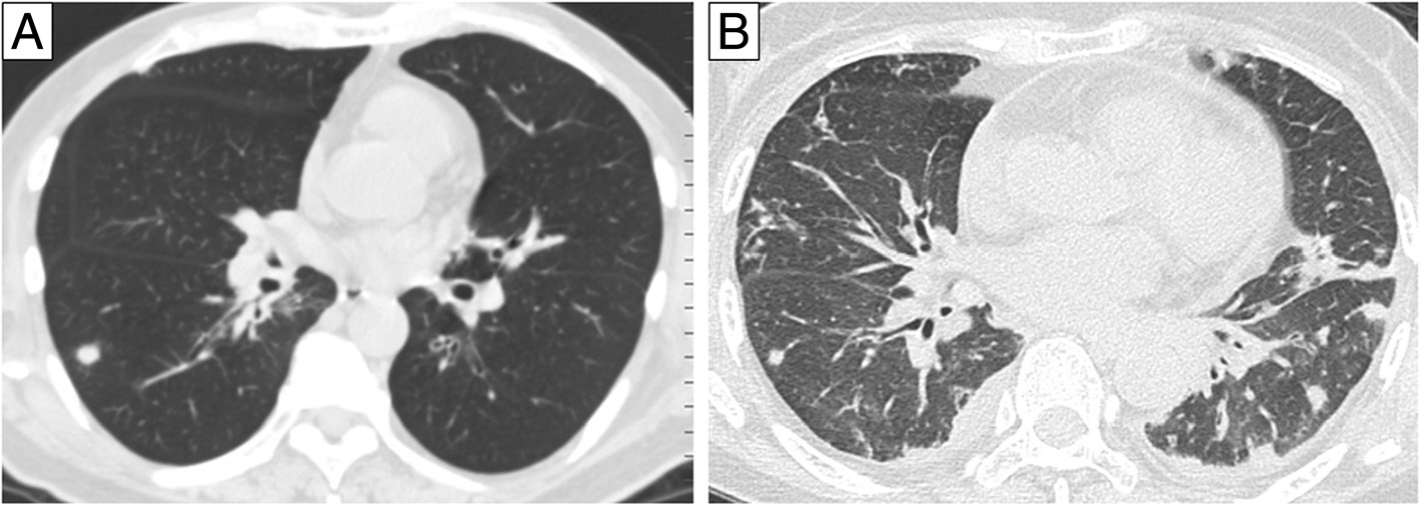
Representative photographs of computed tomography (CT) scans. **a** Initial CT scan of 63 y.o. male positive for PR3-ANCA shows a solitary nodule and wall thickning of lobar bronchi. **b** Initial high-resolution CT scan of 66 y.o female positive for MPO-ANCA shows several nodules, ground glass opacity, wall thickning of lobar and segmental bronchi, and pleural effusion

Consolidation and centrilobular nodular shadow was frequently observed in PR3-ANCA positive cases (56 % and 22 %, respectively). All the other findings, such as ground glass opacity, thickening of interlobular septa, lymphadenopathy, and pleural effusion, were more frequently observed in MPO-ANCA positive cases (67 %, 33 %, 50 %, and 33 %, respectively) (Fig. 2).

### Biopsy sections and histological findings

Biopsy of nasal mucosa, lung, and kidney was performed from 16 sections in eight patients of PR3-positive group and five sections in three patients of MPO-ANCA positive group (Table 4). The most common biopsy section was lung (11 specimens), followed by nasal mucosa (seven specimens), and kidney (three specimens).

**Table 4:** Histopathological findings

	PR3-ANCA	MPO-ANCA
	(N = 9)	(N = 6)
Nasal mucosa biopsy	6	1
Granuloma of artery/perivascular area	2	-
Necrotizing vasculitis	1	-
No siginificant findings	4	1
Lung biopsy	7	4
Granulomatous inflammation of artery/perivascular area	3	1
Necrotizing vasculitis	2	0
Granulomatous inflammation of respiratory tract	3	0
Vasculitis (without necrosis)*	1	1
No siginificant findings	4	2
Renal biopsy	3	0
Granuloma	1	-
Necrotizing vasculitis/glomerulonephritis	2	-
Vasulitis/glomerulonephritis (without necrosis)*	1	-
No siginificant findings	-	-
Classification for ANCA-associated glomerulonephritis		
Cresentic	2	-
Sclerotic	1	-

The specimens of nasal mucosa, lung, and kidney were histopathologically reviewed according to the items of ACR classification criteria and CHCC classification. *; Meaningful but not diagnostic finding. Glomerulonephritis were classified into the categories of focal, crescentic, mixed, or sclerotic according to the histopathologic classification of ANCA-associated glomerulonephritis proposed by Berden.

Granuloma or granulomatous inflammation of artery/perivascular area, which is one of the items of ACR criteria, was detected in six sections (three of the seven specimens of the lung, two of the six specimens of the nasal mucosa, and one of the three specimens of the kidney) in five cases in the PR3-ANCA group, whereas one section (one of four specimens of the lung) was noted in one case in the MPO-ANCA positive group.

Necrotizing vasculitis/glomerulonephritis was detected in five sections (two of the seven specimens of the lung, one of the six specimens of the nasal mucosa, and two of the three specimens of the kidney) in only five cases in the PR3-ANCA group. Glomerulonephritis without necrosis was also detected in one PR3-ANCA positive case. Granulomatous inflammation of the respiratory tract was detected in three sections (three of the seven specimens of the lung) in only three cases in the PR3-ANCA group. Finally, only three of thePR3-ANCA positive cases met the CHCC definition.

According to the histopathologic classification of ANCA-associated glomerulonephritis proposed by Berden [10], two of three cases were classified into the crescentic category (with 50 % of glomeruli with cellular crescents), whereas one case was classified into the sclerotic category (50 % globally sclerotic glomeruli).

### Treatment and outcomes

Combination therapy with prednisone and cyclophosphamide was the most common initial treatment in both groups (67 % for each) (Table 5). Response to the initial treatment was observed in all cases except one. The refractory case also improved after adding rituximab.

**Table 5:** Treatment and outcomes

	PR3-ANCA	MPO-ANCA
	(N = 9)	(N = 6)
Initial treatment		
Prednisone + Cyclophosphamide + Hemodialysis	1	0
Prednisone + Cyclophosphamide	5	4
Prednisone + Methotrexate	1	0
Prednisone + Hemodialysis	1	0
Prednisone	1	2
Additonal treatment		
Cyclophosphamide	0	1
Azathioprine	1	3
for meintenance therapy	0	3
for uncontrolled disease activity	1	0
Methotrexate	1	0
Ritiximab	2	0
Plasma pheresis	1	0
Outcome		
death	1	1
relapse	5	1
alveolar hemorrhage	1	0

The details of initial treatment, additional treatment, and outcomes for the patients in each group were specified

After the induction of remission, the dose of prednisone was decreased gradually; however, relapse was observed in 56 % (5/9) of PR3-ANCA positive cases and 17 % (1/6) of MPO-ANCA positive cases. Among six relapsed cases, the initial medication included prednisone monotherapy (three cases; recurrence rate 100 %), combination with prednisone and methotrexate (one case; recurrence rate 100 %), and combination with prednisone and cyclophosphamide (two cases; recurrence rate 20 %).

During the course of the treatment, cavitation of nodular shadow was observed only in four areas (all cases were PR3-ANCA positive). Alveolar hemorrhage was observed as a manifestation of relapse in one patient in the PR3-ANCA group; however, this improved after the combination therapy with prednisone, cyclophosphamide, and plasmapheresis.

There were only two fatal cases during the observation periods (one case for each group). The direct causes of death were dilated cardiomyopathy and aspiration pneumonia.

## Discussion

In the EULAR recommendation, AAV is defined as chronic inflammatory disease which lasts for more than four weeks, where infection and malignant tumors are excluded, and characteristic histological findings are observed in biopsy or an ANCA-positive result is obtained [11]. AAV includes MPA, GPA, and EGPA; however, the proportion of these disorders differs greatly between Europe/US and Japan [12]. The majority of the AAV patients in Europe and US have GPA, and 80 to 90 % of GPA patients have PR3-ANCA [13]. In contrast, the prevalence of MPA has been reported to be much higher than GPA in Japan. In addition, more than 80 % of Japanese AAV patients were MPO-ANCA positive [14]. These epidemiological differences became the background for the establishment of EMA algorithm [4].

In the present study, 56 % of GPA patients were positive for PR3-ANCA, 38 % were positive for MPO-ANCA, and the remaining 6 % were positive for both. According to the retrospective studies regarding Japanese GPA patients diagnosed by EMA algorithm, the percentage of PR3-ANCA positive patients was 39.5–58.3 % and MPO-ANCA was 33.3–54.6 % [15–17]. High prevalence of MPO-ANCA positive GPA in Japanese was consistently noted, whereas the proportion of MPO-ANCA positive patients was 2.6–13 % in Europe [18, 19]. These differences may be due, in part, to genetic factors. A genome-wide association study of AAVs in European Caucasian patients reported that PR3-ANCA was associated with HLA-DP and genes encoding α1-antitrypsin (SERPINA1) and proteinase 3 (PRTN3), whereas MPO-ANCA was associated with HLA-DQ [20]. Among these, the prevalence of HLA-DPB1*0401 allele was higher in patients with PR3-ANCA associated vasculitis than in patients with MPO-ANCA associated vasculitis or healthy controls. This allele is also less frequent in Japan, China and US African Americans; these are three populations where PR3-ANCA vasculitis is less common than in Europe [21]. In addition, Watts et al. reported that HLA-DPB1*0401 allele frequencies was associated with GPA incidence, and may help explain variations in GPA incidence between populations [22].

With regard to the baseline characteristics, the MPO-ANCA positive group included a greater number of females (67 %). Many studies of GPA showed a male predominance or almost equal numbers of males and females [1, 18, 19, 23]. However, according to the retrospective multi-center study of Japanese patients with GPA or MPA [16], 82 % (14/17) of MPO-ANCA positive GPA were female, a significantly greater population than for PR3-ANCA. Another retrospective study of 24 Japanese patients with GPA also showed that 87.5 % were female [15], consistent with the results of our study.

In the PR3-ANCA positive cases, the time from onset to first visit was significantly shorter than the MPO-ANCA positive cases. On the other hand, although not statistically significant, markers of inflammatory reaction tended to be higher in MPO-ANCA positive cases. No meaningful difference was observed in hemoglobin or serum albumin, which reflects the degree of exhaustion. In addition, there were no significant differences in BVAS. Thus, it cannot be stated which group had higher disease activity and severity.

At the onset of GPA, respiratory tract involvement is usually the most prevalent sign. Patients with GPA have either upper or lower respiratory tract involvement and majority of patients have both [1, 18, 19, 23, 24], whereas in patients with MPA, upper respiratory tract involvement does not occur and pulmonary involvement is usually manifested by alveolar hemorrhage. Characteristics of respiratory tract involvement in MPO-ANCA positive GPA have not been fully clarified until now. In the present study, high frequency of upper respiratory tract involvement was also noted in MPO-ANCA positive cases (67 %) as well as PR3-ANCA positive cases (89 %). As for BVAS at the time of diagnosis, neither total scores nor the scores for every internal organ differ between PR3-ANCA positive cases and MPO-ANCA positive cases. In a retrospective study of 24 Japanese cases of GPA, MPO-ANCA positive cases had nose and sinus involvement less frequently compared to PR3-ANCA positive cases [15]. On the contrary, retrospective multi-center study of Japanese patients with GPA or MPA showed that MPO-ANCA positive cases tended to have ear involvement more frequently, reflected in the fact that otitis media was significantly higher than in PR3-ANCA positive cases [16]. However, neither of the tendencies was observed in the present study, indicating that further investigation is required.

With regard to chest CT imaging, no significant differences were observed in the findings between PR3-ANCA positive cases and MPO-ANCA positive cases. Lohrmann et al. investigated CT images for 57 cases of Wegener’s granulomatosis and reported that the most frequently observed finding is nodular shadow at 89 %, followed by thickening of bronchial walls at 56 % [25]. However, to the best of our knowledge, no report has presented the details of radiological findings in MPO-ANCA positive GPA or compared them with those of PR3-ANCA positive cases. Incidence of nodular shadows (78 % in PR3-ANCA positive cases and 100 % in MPO-ANCA positive cases) and thickening of trachea or bronchial walls (56 % in PR3-ANCA positive cases and 67 % in MPO-ANCA positive cases) reported in this study is close to the results reported in the previous studies of GPA. There were no significant difference in the median number of small/large nodules per person between PR3-ANCA positive cases and MPO-ANCA positive cases.

On the other hands, less common findings including ground glass opacity, thickening of interlobular septa, lymphadenopathy, and pleural effusion, were more frequently observed in MPO-ANCA positive cases in the present study. In addition, bronchial wall thickning from the main bronchi level to the segmental/sub-segmental bronchi level tended to be more conspicuous in MPO-ANCA positive cases. Further investigation is required on whether these differences were accidental occurrence due to the small sample number.

One report noted that cavitation is observed in approximately 30 to 50 % of nodules [26]; however, in the present study the incidence of cavitation was found to be low. In some cases, cavities were formed during the course of the treatment, suggesting that a certain period of time is required for cavities to form in the nodular shadows. It is also possible that as a result of using EMA algorithm, the disorder is discovered at an early stage, before the cavities are formed.

With respect to biopsy sections and histological findings, the detection rate of diagnostic findings, such as granuloma/granulomatous inflammation of an artery/perivascular area, necrotizing vasculitis/glomerulonephritis, granulomatous inflammation of the respiratory tract, was the highest for biopsy of the kidney (66 %), followed by the lung (40 %) and nasal mucosa (29 %). Significant findings leading to the diagnosis of GPA are rarely seen in specimens from the upper respiratory tract, as previously reported [27–29]. For lung biopsy, the detection rate of diagnostic findings was 100 % for cases where video-assisted thoracic surgery (VATS) was performed. TBLB or echo/CT-guided biopsy resulted in a lower detection rate (20 %, 33 %, and 0 %, respectively). Meaningful but not diagnostic findings such as vasculitis without necrosis were observed in small specimens by TBLB and nasal mucosa. These results suggest that sufficient tissue is necessary for successful pathological verification.

However, histopathological investigation cannot be conducted in some cases; thus, establishment of useful surrogate marker is desired. In this study, the most common surrogate markers defined in the EMA algorithm were fixed pulmonary infiltrates, nodules, or cavitation present for >1 month (78 % in PR3-ANCA positive cases and 86 % in MPO-ANCA positive cases, respectively), followed by bronchial stenosis (33 % and 50 %, respectively) and chronic sinusitis, otitis media, or mastoiditis for >3 months (56 % and 67 %, respectively) (Table 6). The detection rate of these markers in MPO-ANCA positive cases was almost the same as that of PR3-ANCA positive cases. Thus, investigation of respiratory tract involvement according to these surrogate markers will assist the diagnosis of GPA in the cases without histological proof of granuloma/necrotizing vasculitis even in MPO-ANCA positive cases.

**Table 6:** Details of the items applied to each patient according to the EMA algorithm

		PR3-ANCA (N = 9)	MPO-ANCA (N = 6)	PR3 + MPO (N = 1)
ACR criteria				
1 item	abnormal chest radiograph	0	3 (50 %)	0
2 items	purulent/bloody nasal discharge + granuloma formation	2 (22 %)	1 (17 %)	0
	abnormal chest radiograph + abnormal urinary sediment	1 (11 %)	1 (17 %)	1 (100 %)
3 items	purulent/bloody nasal discharge + abnormal chest radiograph + granuloma formation	3 (33 %)	0	0
	abnormal chest radiograph + abnormal urinary sediment + granuloma formation	1 (11 %)	0	0
	purulent/bloody nasal discharge + abnormal chest radiograph + abnormal urinary sediment	2 (22 %)	1 (17 %)	0
CHCC criteria				
0 item		4 (44 %)	6 (100 %)	1 (100 %)
1 item	granuloma formation	0	0	0
	necrotizing vasculitis	2 (22 %)	0	0
2 items	granuloma formation + necrotizing vasculitis	3 (33 %)	0	0
Surrogate marker				
fixed pulmonary infiltrates, nodules, or cavitations (>1 month)		7 (78 %)	5 (86 %)	1 (100 %)
bronchial stenosis		3 (33 %)	3 (50 %)	1 (100 %)
bloody nasal discharge and crusting (>1 month), or nasal ulceration		4 (44 %)	0	0
chronic sinusitis, otitis media, or mastoiditis (>3 months)		5 (56 %)	4 (67 %)	1 (100 %)
retro-orbital mass or inflammation (pseudotumour)		2 (22 %)	0	0
saddle nose deformity/destructive sinonasal disease		1 (11 %)	0	0

Abbreviations: *EMA* European Medicine Agency, *ACR* American College of Rheumatology, *CHCC* Chapel Hill Consensus Conference

The prognosis of AAV markedly improved with the combination therapy of high-dose steroids and cyclophosphamide [4]. In the present study, all the patients initially treated with prednisone monotherapy or combination with prednisone and methotrexate relapsed during the treatment course. On the other hand, the rate of relapse was only 20 % in patients initially treated with prednisone and cyclophosphamide.

In addition, the recent randomized trial of rituximab showed that rituximab therapy was superior to cyclophosphamide treatment in relapsing cases of AAV [30]. In the present study, rituximab was administered in two cases; one case was refractory to the initial combination therapy with prednisone and cyclophosphamide, and the other case was refractory to prednisone and cyclophosphamide started after relapse. Both cases improved immediately by addition of rituximab, and no relapse has occurred as of this writing. Moreover, rituximab was as effective as continuous conventional immunosuppressive therapy in the patients with severe AAV [31]. In future, initial intensive treatment including rituximab may lead to favorable prognosis.

In the present study, the rate of relapse in MPO-ANCA positive cases was lower than that of PR3-ANCA positive cases (17 % and 56 %, respectively). Relapses are more common in patients with GPA (25 to 80 % of patients) than in those with MPA, in whom relapse has been reported in 8 % at 18 months [32]. According to the community-based cohort study of 350 patients with newly diagnosed AAV, the positivity for PR3-ANCA and involvement of the lung and the upper respiratory tract were associated with relapse [33]. Even among patients of GPA, MPO-ANCA positive cases may be less likely to relapse than PR3-ANCA positive cases.

Limitations of the present study include the small number of patients investigated and possible deviation of patient distribution. Another limitation is insufficient histopathological investigation in MPO-ANCA positive cases. The short observation period is also a problem when assessing long-term prognosis.

## Conclusions

In the present study, a high prevalence of MPO-ANCA positive GPA was consistently noted, which is higher in Japan than in Europe or the US. No significant differences of clinico-radiological findings were observed except for the prevalence of relapse between the PR3-ANCA positive cases and MPO-ANCA positive cases, suggesting that the type of ANCA may be of little help to the diagnosis of GPA. Examination for granulomatous findings in the upper and lower respiratory tract is very important even in MPO-ANCA positive cases. There is a need to accumulate more cases and conduct a further investigation in the future.

## Abbreviations

GPA: Granulomatosis with polyangiitis
ACR: American College of Rheumatology
CHCC: Chapel Hill Consensus Conference
EMA: European Medicine Agency
ANCA: Antineutrophil cytoplasmic antibody
AAV: ANCA-associated vasculitis
PN: Polyarteritis nodosa
EULAR: European League Against Rheumatism
PR3: Proteinase 3
MPO: Myeloperoxidase
MPA: Microscopic polyangiitis
EGPA: Eosinophilic granulomatosis with polyangiitis
BVAS: Birmingham Vasculitis Activity Score
CT: Computed tomography
